# Birth weight, maternal weight and childhood leukaemia

**DOI:** 10.1038/sj.bjc.6603173

**Published:** 2006-05-30

**Authors:** C C McLaughlin, M S Baptiste, M J Schymura, P C Nasca, M S Zdeb

**Affiliations:** 1 New York State Cancer Registry, New York State Department of Health, Corning Tower Room 536, Empire State Plaza, Albany, NY 12237-0679, USA; 2 State University of New York at Albany School of Public Health, Albany, NY, USA; 3 Division of Chronic Disease Prevention and Adult Health, New York State Department of Health, Albany, NY, USA; 4 Department of Public Health, University of Massachusetts at Amherst School of Public Health and Health Sciences, Amherst MA, USA

**Keywords:** leukaemia, birth weight, maternal weight, pregnancy weight gain, maternal age

## Abstract

There is mounting evidence that childhood leukaemia is associated with high birth weight, but few studies have examined the relationship between leukaemia and other perinatal factors that influence birth weight, such as maternal weight or gestational weight gain. This case-cohort study included 916 acute lymphocytic leukaemia (ALL) and 154 acute myeloid leukaemia (AML) cases diagnosed prior to age 10 years between 1985 and 2001 and born in New York State excluding New York City between 1978 and 2001. Controls (*n*=9686) were selected from the birth cohorts for the same years. Moderate increased risk of both ALL and AML was associated with birth weight 3500 g or more. For ALL, however, there was evidence of effect modification with birth weight and maternal prepregnancy weight. High birth weight was associated with ALL only when the mother was not overweight while heavier maternal weight was associated with ALL only when the infant was not high birth weight. Increased pregnancy-related weight gain was associated with ALL. For AML, birth weight under 3000 g and higher prepregnancy weight were both associated with increased risk. These findings suggest childhood leukaemia may be related to factors influencing abnormal fetal growth patterns.

Leukaemia is the most common form of childhood cancer. There is strong evidence that at least some childhood leukaemias that arise before the age of 14 years have prenatal origins (Greaves *et al*, 2003), but despite a growing understanding of the molecular carcinogenic process, the risk factors for the pre- and postnatal DNA mutations associated with leukaemia are not known. There is mounting evidence that higher birth weight is associated with childhood leukaemia. A recent meta-analysis of birth weight and leukaemia found approximately 25% increased risk of both acute lymphocytic leukaemia (ALL) and acute myeloid leukaemia (AML) with birth weight over 4000 g, although the studies of AML showed more heterogeneity than the studies of ALL (Hjalgrim *et al*, 2003). Other birth-related factors for which the association with ALL is suggestive include older maternal age and history of fetal loss prior to index birth (Little, 1999). Maternal subfertility has been suggested as a potential factor in the aetiology of ALL (Steensel-Moll *et al*, 1985). Being the first-born or only child and higher socioeconomic status has also been associated with increased risk of ALL, which Kinlen (1988) and Greaves *et al* (2003) hypothesised is due to differential risk of age-specific exposure to viruses. For AML, there have been several studies suggesting associations with maternal alcohol consumption during pregnancy and parental exposure to pesticides or benzene (Little, 1999).

We used existing public health surveillance data to perform a case-cohort study of leukaemia and perinatal risk factors. We hypothesised that high birth weight, first born status, older maternal age and history of prior fetal loss would be associated with the risk of both ALL and AML. The data collected on the New York State birth certificate also allowed for exploratory analysis with respect to factors associated with birth weight and fetal growth. In addition to birth weight and gestational age, several risk factors for macrosomia are available from the New York State birth records, including maternal diabetes, multiparity, prior history of a macrosomic infant, postdate gestation, maternal weight, and pregnancy weight gain.

## MATERIALS AND METHODS

Leukaemia cases were ascertained from the New York State Cancer Registry. All cases age 1 month to 9 years diagnosed with acute leukaemia between 1985 and 2001 while resident of New York State and born in New York State excluding New York City were eligible to be included in the study. Children with leukaemia diagnosed subsequent to another form of cancer were excluded. Eligibility was determined by matching the leukaemia cases to the electronic birth files of children born in New York State between 1978 through 2001. Because the birth certificate used in New York State differs from the certificate used in New York City and other states, children who were found to have been born in New York City or out-of-state were excluded, as were children who were not matched to electronic birth certificate records. Leukaemias were grouped into ALL and AML based on the ICD-O-3 version of the International Classification of Childhood Cancers (Steliarova-Foucher *et al*, 2005). We excluded 79 matched leukaemia cases who could not be classified as either ALL or AML, including 35 children with unspecified types of leukaemia and 44 children with chronic or other specified leukaemia. Immunophenotype was not available for 85% of ALL cases, and was therefore not included in the analysis. One case was diagnosed simultaneously with both ALL and AML, and is included in the analyses for both outcomes.

Controls were selected from electronic birth files of all children whose place of birth was New York State excluding New York City between 1978 and 2001. This study was a subset of a larger study, which included childhood cancer cases for all cancer sites. Two controls were selected for each cancer case included in the larger study, resulting in 9686 controls. As the data elements available from the birth records vary by year of birth, controls were frequency matched to the cases included in the larger study based on year of birth to assure consistency of availability of information. For the purposes of individual cancer types such as reported here, frequency of birth year was not necessarily proportional between cases and controls. One ALL case was also selected as a control, and was retained in the analysis as both a case and a control. Additionally, 13 controls were also in the registry-birth match and therefore were known to have developed other forms of cancer or benign brain tumours. Children who died in the neonatal period (within 28 days of birth) were also excluded, because cases diagnosed at less than 1 month of age were excluded from the case series.

The independent variables for this study came from the birth certificates of the cases and controls. The birth certificates are completed by the staff at the hospital where the child is born. Data from the birth certificates are routinely abstracted and entered onto the electronic master live birth file, and are available for the birth cohorts included in this study (1978–2001). The birth certificates underwent major revisions in 1988 and 1993. In addition to changes in the birth certificate itself, the data items available on the master birth file have also changed, as has the coding of individual variables. Owing to these changes, some independent variables were not available for analysis for all cases. For example, maternal height was not collected on the New York State birth certificates until 1993, and therefore maternal body mass index could not be calculated for 75% of leukaemia cases and 81% of controls. Maternal prepregnancy weight was collected starting in 1988, and was missing for a similar proportion of cases and controls born between 1998 and 2001 (7.6 and 8.0%, respectively). Maternal weight and weight gain were recorded in pounds on the birth certificate and converted to kilograms for this analysis. There was evidence of end-digit preference in reporting weight gain in pounds, with modes at 20, 25, 30 and 40 pounds. We used these modes for cutpoints in the analysis, which resulted in unusual weight gain categories when reported in kilograms.

This study design was a case-cohort study; therefore, the odds ratio provides an estimate of the cumulative incidence (risk) ratio (Rothman and Greenland, 1998). We defined high birth weight as 3500 g or more, very high birth weight as 4500 g or more, and low birth weight as less than 2500 g. We used 80 kg to indicate heavier maternal weight, which corresponds to obesity for an average height woman (body mass index of 30 for a woman of 163 cm), but would be considered normal weight for a tall woman (body mass index 23.7 for a woman 180 cm). Separate analyses were conducted for ALL and AML. For ALL, additional analyses were conducted stratified by age at diagnosis. Unconditional logistic regression was used for multivariate analysis. As some exposures changed prevalence over the course of the study period, birth year, as a continuous variable, was included in all regression models. Unless otherwise noted, all models included birth year, birth weight, gender, gestational age, maternal age, race and ethnicity.

## RESULTS

### Acute lymphocytic leukaemia

There were 1102 ALL cases diagnosed in New York State excluding New York City who were eligible to be included in the study. Birth certificate matches were made for 916 cases, resulting in a match proportion of 83%. The estimated match proportion was slightly higher for cases diagnosed ages 0–4 years (87%) compared to cases diagnosed 5–9 years of age (76%).

The birth weight distribution of ALL cases and controls is presented in Table 1, with stratification by age at diagnosis. High birth weight was associated with increased risk of ALL. The adjusted odds ratio for birth weight of 3500 g or more, compared to birth weight under 3500 g, was 1.17 (95% confidence interval 1.01, 1.35). In addition, lower birth weights were associated with decreased risk. The odds ratio for birth weight under 3000 g was 0.76 (95% confidence interval 0.61, 0.94), relative to 3000–3499 g. The test for trend for increasing ALL risk with increasing birth weight was statistically significant (*P*=0.0015), and the adjusted odds ratio associated with a 1000 g increase in birth weight as a continuous variable was 1.25 (95% confidence interval 1.08, 1.43). This association did not vary by age. The adjusted odds ratio for high birth weight for ALL diagnosed within the first 4 years of life was 1.18 (95% confidence interval 0.99, 1.40), while the adjusted odds ratio for diagnosis at ages 5 to 9 years was 1.15 (95% confidence interval 0.90, 1.48). The test for trend, however, was not statistically significant for ALL diagnosed at ages 5–9 years.

Maternal prepregnancy weight was not statistically significantly associated with increased risk of ALL (Table 1), although there was evidence of a dose response, with a significant test for trend for ALL at all ages (*P*=0.03) and for diagnosis prior to the age of 5 years (*P*=0.002). Body mass index classified as overweight or obese (>25) was associated with an adjusted odds ratio of 1.44 (95% confidence interval 1.03, 2.01) for ALL diagnosis prior to the age of five, relative to body mass index of 20–24. Since maternal height was not collected prior to 1993, maternal body mass index was not available for the ALL diagnosed at an older age. There was evidence of interaction between maternal prepregnancy weight and ALL as well (Table 2). High birth weight and heavier maternal weight were themselves associated; among controls, the adjusted odds ratio for the risk of high birth weight associated with maternal weight over 80 kg was 1.85 (95% confidence interval 1.54, 2.23). We observed increased risk of ALL among children whose births weights were discordant with the mother's weight. That is, increased risk was observed among children who were heavier at birth but whose mothers were not heavy, as well as among children without high birth weight but whose mothers were heavier. Conversely, increased risk was not observed among children whose birth weights were concordant with maternal weight, indicating antagonistic effect modification. The interaction term in the logistic regression model for high birth weight and heavier maternal weight as dichotomised in Table 2 was statistically significant (*P*=0.006). These results varied slightly by age at diagnosis, although the number of cases became sparse (Table 2). For ALL diagnosed between the ages of 5 and 9 years, the combination of high birth weight and high maternal weight was protective, but only two cases in the age group with this combined exposure were observed.

Higher maternal weight gain during pregnancy was also associated with ALL (Table 1), and there was no evidence of interaction with birth weight or maternal weight in the logistic regression model (*P*=0.14). The pattern of increased risk with more weight gain was consistent across the two age groups examined, although the odds ratios did not reach statistical significance for leukaemia diagnosed at ages 5–9 years. When dichotomised into weight gain of more than 14 kg, relative to <14 kg, the adjusted odds ratio for ALL was 1.31 (95% confidence interval 1.07, 1.60). For ALL diagnosed prior to the fifth birthday, the adjusted odds ratio was 1.26 (95% confidence interval 1.00, 1.59) and for ages 5–9 it was 1.46 (95% confidence interval 1.01, 2.14).

Maternal diabetes was associated with nonsignificant increased risk of ALL after controlling for the other weight factors. For ALL diagnosed prior to the age of 5 years, older maternal age was associated with increased risk, but no increased risk was observed for older age at diagnosis. Other factors examined, but which were not associated with risk of ALL in the multivariate analysis include paternal age, first born status, number of previous live births, maternal history of prior fetal loss, publicly funded financial assistance during pregnancy, method of delivery or use of obstetric procedures during pregnancy and delivery, infertility treatment, maternal smoking or drug use, other maternal medical risk factors such as hypertension, or infant medical risk factors such as birth injury or assisted ventilation (data not shown).

### Acute myeloid leukaemia

There were 186 AML cases eligible to be in the study, of whom 154 matched to birth records, resulting in an estimated match proportion of 83%. The cell types of AML included in this study included AML, not otherwise specified (96 cases), acute promyelocytic leukaemia (12 cases), acute myelomonocytic leukaemia (15 cases), acute monocytic leukaemia (16 cases), acute megakaryoblastic leukaemia (11 cases) and four cases with other AML cell types.

Both low birth weight and high birth weight were associated with increased risk of AML, as was heavier prepregnancy weight (Table 1). Owing to the small number of AML cases, we also analysed birth weight with wider categories. The odds ratio for birth weight under 3000 g, relative to 3000–3499 g, was 1.80 (95% confidence interval 1.11, 2.59); for birth weight over 3500 g the odds ratio was 1.76 (1.18, 2.68). Unlike ALL, however, there was no evidence of interaction between birth weight and maternal weight in the logistic regression model (*P*=0.41). Additionally, higher weight gain during pregnancy was not associated with AML. In fact, there was a trend towards decreasing risk with increasing weight gain, and the test for trend was borderline statistically significant (one-sided *P* for trend=0.046). Older maternal age was also associated with increased risk of AML. The adjusted odds ratio for maternal age 40 years or older, relative to 20–29 years of age, was 3.68 (95% confidence interval 1.48, 7.85). As with ALL, we did not observe associations between AML and the other maternal or infant risk factors examined (data not shown).

## DISCUSSION

A recent meta-analysis of 18 studies from the 1940s onward found higher risk of ALL with birth weight of over 4000 g (pooled odds ratio of 1.26, 95% confidence interval 1.17–1.37) (Hjalgrim *et al*, 2003). In addition, risk increased 14% with each 1000 g increase in birth weight. While some studies have been negative (Robison *et al*, 1987; Kaye *et al*, 1991; Savitz and Ananth, 1994; Dockerty *et al*, 1999; Thompson *et al*, 2001; Ma *et al*, 2005), the majority of studies support a weak to moderate increased risk of ALL among children who had heavier birth weights (Buckley *et al*, 1994; Cnattingius *et al*, 1995; Ross *et al*, 1997; Westergaard *et al*, 1997; Yeazel *et al*, 1997; Smulevich *et al*, 1999; Suminoe *et al*, 1999; Murray *et al*, 2002; Okcu *et al*, 2002; Reynolds *et al*, 2002; Shu *et al*, 2002), including some studies published since the meta-analysis (Hjalgrim *et al*, 2004; Jourdan-Da Silva *et al*, 2004; Lee *et al*, 2004; Paltiel *et al*, 2004). Our findings with respect to birth weight are consistent with previous studies. The presence of possible interaction with maternal weight, however, suggests that the relationship between birth weight and ALL may be more complex than previously described. In our results, birth weight was associated with increased risk of ALL only when the mother's weight was not heavy. Conversely, children born to heavier mothers but whose birth weights were not high were also at increased risk. Children with high birth weight concordant with high maternal weight were not at increased risk of ALL. This antagonistic effect modification supports the previously suggested hypothesis that factors related to prenatal growth, rather than birth weight *per se*, are involved in the mechanism by which birth weight is associated with ALL. These findings warrant further study, since our evaluation of this interaction was exploratory in nature, and limited to births occurring between 1988 and 2001.

In Hjalgrim's meta-analysis, the point estimate of the risk ratio for birth weight and AML was of similar magnitude as for ALL, although not statistically significant (pooled odds ratio 1.27, 95% confidence interval 0.73–2.20) and 29% increase in risk per 1000 g increase in birth weight. For AML, however, there was evidence of heterogeneity, with two studies showing a nonsignificant protective effect of high birth weight (Shu *et al*, 1999; Reynolds *et al*, 2002) and two studies showing a statistically significant elevated relative risk (Westergaard *et al*, 1997; Suminoe *et al*, 1999). In our study, we did not observe a dose response for birth weight and AML. Rather, AML was associated with both low birth weight and high birth weight. The U-shaped dose–response curve for birth weight and AML was also reported in a recent large study from Scandinavia (Hjalgrim *et al*, 2004). Most studies that have reported on AML and birth weight in ordinal categories have not yielded similar results (Shu *et al*, 1988; Ross *et al*, 1997; Yeazel *et al*, 1997; Shu *et al*, 1999; Reynolds *et al*, 2002; Jourdan-Da Silva *et al*, 2004), although several used relatively high cutoffs (2700 or 3000 g) for the lowest birth weight category (Shu *et al*, 1988; Ross *et al*, 1997; Yeazel *et al*, 1997; Shu *et al*, 1999). The presence of a nonlinear effect would explain the heterogeneity among studies in the meta-analysis.

A number of mechanisms have been suggested to explain the observed association between birth weight and leukaemia, including insulin-like growth factor I (IGF-I), which is associated with higher birth weight and hypothesised to stimulate myeloid and lymphoid cells (Ross *et al*, 1996), and decreased thyroid function in the prenatal period, which is inversely correlated with birth weight and is hypothesised to be associated with leukaemia by leading to decreased cell turn over or by a direct affect on lymphocytes (Lei *et al*, 2000).

Birth weight is a complicated outcome, which is itself associated not only with gestational age and infant health but also with a number of maternal risk factors, including weight and weight for height, pregnancy weight gain, multiparity, diabetes, race and ethnicity, diet, smoking and other drug use, and the mother's own birth weight (Skjaerven *et al*, 1997; Orskou *et al*, 2003; Ehrenberg *et al*, 2004; Clausen *et al*, 2005). These factors are all potentially related to leukaemia through uncontrolled confounding, independent affects, effect modification, or by acting along the same causal pathway. We were able to explore a number of these factors in this study, and, in addition to prepregnancy weight, we observed increased risk of ALL with pregnancy weight gain, but no association with multiparity, diabetes, smoking or history of previous high birth weight infants. Obesity and excessive weight gain can cause hyperglycemia in both the mother and infant. Exposure to hyperglycemia would increase the infant's production of insulin, and the possibility of exposure to hyperglycemia during pregnancy and increased risk of childhood and adult chronic diseases associated with impaired glucose metabolism is under study (Boney *et al*, 2005). Concerning the antagonistic effect modification between birth weight and maternal weight observed in this study, several studies have observed effect modification between with maternal weight and markers of insulin metabolism in relation to macrosomia (Jaksic *et al*, 2001; Ehrenberg *et al*, 2004; Clausen *et al*, 2005). For example, Clausen *et al* (2005) hypothesised that slim women with large babies may have higher levels of insulin resistance compared to heavier mothers with large babies. Ehrenberg *et al* (2004) reported that the influence of diabetes on birth weight was stronger for normal and underweight women, compared to overweight women. Chronic (preconception) diabetes and maternal obesity may also introduce additional maternal medical risk factors such as cardiovascular problems and nephropathy (Boulet *et al*, 2003). Maternal obesity could also be associated with increased endogenous hormones and toxin buildup in adipose tissue.

We also observed increased risk of both ALL and AML with older maternal age, although the results were stronger for AML and for ALL diagnosed under age 5 years. The hypothesis that older maternal age may be associated with childhood leukaemia arises from the joint fact that older maternal age is associated with increased risk of chromosomal malformations, and the fact that childhood leukaemia is a disease related to chromosomal breakage and translocation. A few earlier studies published in the 1960s or early 1970s suggested a weak increased risk of leukaemia or leukaemia death among offspring born to older mothers in the United States (Manning and Carroll, 1957; MacMahon and Newill, 1962; Stark and Mantel, 1969; Fasal *et al*, 1971). Since then, however, most studies of maternal age and the risk of leukaemia in the offspring have been negative or have shown weak, nonsignificant elevated risk with older maternal age (Salonen and Saxen, 1975; Kneale and Stewart, 1976; Shaw *et al*, 1984; Steensel-Moll *et al*, 1985; McKinney *et al*, 1987, 1999; Shu *et al*, 1988, 1994, 1999; Zack *et al*, 1991; Cnattingius *et al*, 1995; Petridou *et al*, 1997; Roman *et al*, 1997; Ross *et al*, 1997; Westergaard *et al*, 1997; Dockerty *et al*, 1999; Thompson *et al*, 2001; Murray *et al*, 2002; Okcu *et al*, 2002; Jourdan-Da Silva *et al*, 2004). A handful of studies have resulted in statistically significant increased risk ratios in the order of 1.5–2.0 for ALL or all leukaemias among children born to women older than age 35 years at delivery (Kaye *et al*, 1991; Buckley *et al*, 1994; Hemminki *et al*, 1999; Mogren *et al*, 1999; Dockerty *et al*, 2001; Reynolds *et al*, 2002; Shu *et al*, 2002; Jourdan-Da Silva *et al*, 2004). Little (1999) proposed that the lack of consistency between the early studies and those conducted later was due to the introduction of easily accessible contraception and abortion, which has introduced the possibility of confounding by socioeconomic status. Among the positive studies, there is no distinct pattern of elevated risk related to maternal age in terms of the child's age at diagnosis, leukaemia subtype or gender.

As a case-cohort study, lack of follow-up of controls creates a potential for selection bias. The potential loss-to-follow-up in this study arises from the fact that controls were ascertained from existing records with no subsequent follow-up (other than eliminating potential controls who died within the first month of life). By sampling the controls from the birth cohorts, we are assuming that the controls had they developed cancer, would have been ascertained as cases in the study, and that no controls leave the risk set due to death. Controls who develop cancer may be missed if they migrate out of New York or, less likely, if they were unreported to the state cancer registry. Childhood mortality is associated, either directly or indirectly, with several of the perinatal factors being studied, including infant birth weight and maternal age. After the immediate postnatal period, childhood mortality is low (National Center for Health Statistics, 1994). Despite these limitations, the use of a case-cohort study design based on cancer registry and birth records has several advantages over a case–control study design based on interviews with respect to selection bias. By using administrative record data instead of interview, there is less potential for self-selection bias related to refusal of cases or controls to participate in the study or selection bias related to inability to trace and contact study subjects.

Use of birth certificate data for ascertaining perinatal exposures reduces the potential for differential misclassification; since all measurement is made prior to diagnosis and measurement does not rely on parental recall of pregnancy history. Birth certificates, however, may be problematic for epidemiologic studies due to problems with completeness and validity (Kirby, 2001). In a study of the validity of electronic birth certificate data for New York State excluding New York City, Roohan observed that the sensitivity of maternal medical risk factors on the birth certificates varied, but specificity was high (Acar *et al*, 2001; Roohan *et al*, 2003). For maternal prepregnancy weight and weight at delivery, the birth certificates were within five pounds of the weight (2.2 kg) recorded in the medical record for over 87% of records sampled (Acar *et al*, 2001). Lack of sensitivity of the birth certificate may also explain why several potential risk factors that have been suggested in previous studies were not confirmed in this study.

By using the birth certificate data, we were able to examine a number of factors related to infant's birth weight, some of which have not been reported on in previous studies. Our findings related to maternal weight and pregnancy weight gain are therefore exploratory, but, given the high prevalence of overweight and obesity, the possible association with childhood leukaemia should be examined in other cohorts. Maternal weight and height, as well as pregnancy weight gain, were added to the United States standard birth certificate in 2003. While the use of the birth certificate for epidemiologic studies has been questioned, the future availability of these data may promote cost-effective, population-based studies of perinatal risk factors for childhood cancer.

## Figures and Tables

**Table 1:** Adjusted odd ratios for other maternal and infant birth characteristics, acute lymphocytic leukaemia (ALL) and acute monocytic leukaemia (AML) children born in New York State excluding New York City, 1978–2000

	**Controls**		**ALL (all ages)**				**ALL age 0–4 years**				**ALL age 5–9 years**				**AML (all ages)**			
	***N***	**(%)**	***N***	**(%)**	**RR^a^**	**(95% CI)**	***N***	**(%)**	**RR^a^**	**(95% CI)**	***N***	**(%)**	**RR^a^**	**(95% CI)**	***N***	**(%)**	**RR^a^**	**(95% CI)**
*Birth weight*																		
<2500 g	587	(6.1)	42	(4.6)	0.73	(0.50, 1.06)	21	(3.3)	0.46	(0.27, 0.75)	21	(7.3)	1.47	(0.84, 2.48)	12	(7.9)	1.94	(0.88, 4.00)
2500–2999 g	1466	(15.2)	107	(11.7)	0.77	(0.61, 0.97)	81	(12.9)	0.85	(0.64, 1.11)	26	(9.1)	0.59	(0.37, 0.91)	26	(17.1)	1.77	(1.04, 2.96)
3000–3499 g	3496	(36.2)	332	(36.2)	1	(reference)	227	(36.0)	1	(reference)	105	(36.7)	1	(reference)	37	(24.3)	1	(reference)
3500–3999 g	2999	(31.0)	313	(34.2)	1.07	(0.91, 1.26)	210	(33.3)	1.06	(0.86, 1.29)	103	(36.0)	1.11	(0.84, 1.47)	51	(33.6)	1.59	(1.03, 2.49)
4000–4499 g	935	(9.7)	101	(11.0)	1.10	(0.86, 1.39)	77	(12.2)	1.22	(0.92, 1.60)	24	(8.4)	0.85	(0.53, 1.32)	18	(11.8)	1.90	(1.04, 3.36)
4500+ g	184	(1.9)	21	(2.3)	1.10	(0.67, 1.73)	14	(2.2)	1.05	(0.57, 1.78)	7	(2.4)	1.26	(0.52, 2.57)	8	(5.3)	3.89	(1.63, 8.26)
																		
*Gender*																		
Male	5069	(52.4)	520	(56.8)	1.18	(1.02, 1.36)	366	(58.1)	1.23	(1.04, 1.46)	154	(53.8)	1.07	(0.84, 1.37)	84	(54.9)	1.14	(0.82, 1.60)
Female	4603	(47.6)	396	(43.2)	1	(reference)	264	(41.9)	1	(reference)	132	(46.2)	1	(reference)	69	(45.1)	1	(reference)
																		
*Race and ethnicity*																		
White non-Hispanic	7326	(75.9)	746	(81.8)	1	(reference)	508	(80.9)	1	(reference)	238	(83.8)	1	(reference)	118	(76.6)	1	(reference)
Black	641	(6.6)	33	(3.6)	0.51	(0.35, 0.72)	23	(3.7)	0.51	(0.32, 0.77)	10	(3.5)	0.51	(0.25, 0.92)	13	(8.4)	0.94	(0.47, 1.71)
Hispanic	1284	(13.3)	92	(10.1)	0.78	(0.61, 0.98)	68	(10.8)	0.89	(0.67, 1.16)	24	(8.5)	0.58	(0.37, 0.89)	19	(12.3)	0.97	(0.56, 1.57)
Other	403	(4.2)	41	(4.5)	0.90	(0.63, 1.26)	29	(4.6)	0.87	(0.57, 1.29)	12	(4.2)	0.98	(0.50, 1.74)	4	(2.6)	0.46	(0.14, 1.13)
																		
*Gestational age*																		
<35 weeks	304	(3.2)	27	(3.0)	1.23	(0.77, 1.89)	21	(3.4)	1.72	(1.01, 2.80)	6	(2.1)	0.59	(0.22, 1.34)	5	(3.3)	0.85	(0.28, 2.15)
35–37 weeks	830	(8.8)	82	(9.0)	1.18	(0.90, 1.51)	56	(8.9)	1.22	(0.89, 1.63)	26	(9.2)	1.09	(0.68, 1.68)	16	(10.6)	1.06	(0.57, 1.85)
38–41 weeks	6505	(68.9)	628	(68.9)	1	(reference)	432	(68.8)	1	(reference)	196	(69.0)	1	(reference)	101	(66.9)	1	(reference)
42+ weeks	1801	(19.1)	156	(17.1)	0.95	(0.78, 1.14)	104	(16.6)	0.95	(0.75, 1.82)	52	(18.3)	0.94	(0.68, 1.28)	29	(19.2)	1.01	(0.64, 1.55)
																		
*Pregnancy weight gain (1988–2001 births only)*																		
<8.6 kg	511	(13.2)	60	(12.8)	1.31	(0.90, 1.91)	43	(12.5)	1.21	(0.78, 1.87)	17	(13.9)	1.59	(0.78, 3.28)	19	(19.4)	1.45	(0.73, 2.90)
9.1–11.3 kg	696	(18.0)	65	(13.9)	1	(reference)	49	(14.2)	1	(reference)	16	(13.1)	1	(reference)	18	(18.4)	1	(reference)
11.8–13.6 kg	749	(19.4)	83	(17.8)	1.12	(0.79, 1.59)	64	(18.6)	1.12	(0.75, 1.67)	19	(15.6)	1.11	(0.56, 2.21)	17	(17.3)	0.90	(0.44, 1.84)
14.1–18.1 kg	1138	(29.5)	158	(33.8)	1.42	(1.04, 1.94)	113	(32.8)	1.30	(0.92, 1.87)	45	(36.9)	1.80	(1.02, 3.34)	25	(25.5)	0.88	(0.47, 1.71)
18.6+ kg	766	(19.8)	101	(21.6)	1.38	(0.99, 1.94)	76	(22.0)	1.35	(0.92, 2.00)	25	(20.5)	1.45	(0.76, 2.82)	19	(19.4)	0.83	(0.41, 1.68)
																		
*Prepregnancy weight (1988–2001 births only)*																		
<56 kg	1102	(28.0)	119	(25.2)	1	(reference)	80	(22.8)	1	(reference)	39	(32.2)	1	(reference)	20	(20.0)	1	(reference)
56–67 kg	1539	(39.1)	183	(38.8)	1.05	(0.82, 1.35)	136	(38.7)	1.15	(0.86, 1.55)	47	(38.8)	0.86	(0.55, 1.34)	42	(42.0)	1.54	(0.89, 2.75)
68–79 kg	775	(19.7)	99	(21.0)	1.18	(0.88, 1.58)	74	(21.1)	1.30	(0.92, 1.83)	25	(20.7)	0.95	(0.56, 1.60)	15	(15.0)	0.89	(0.42, 1.83)
80+ kg	522	(13.3)	71	(15.0)	1.25	(0.89, 1.73)	61	(17.4)	1.54	(1.06, 2.23)	10	(8.3)	0.58	(0.26, 1.17)	23	(23.0)	2.25	(1.18, 4.34)
																		
*Maternal diabetes*																		
Present	164	(1.8)	25	(2.9)	1.44	(0.91, 2.18)	18	(3.0)	1.37	(0.80, 2.21)	7	(2.6)	2.29	(0.86, 5.10)	1	(0.7)	0.26	(0.02, 1.18)
Not recorded	9085	(98.2)	846	(97.1)	1	(reference)	579	(97.0)	1	(reference)	267	(97.4)	1	(reference)	145	(99.3)	1	(reference)
																		
*Maternal age*																		
<20 years	885	(9.1)	66	(7.2)	0.93	(0.69, 1.22)	43	(6.9)	0.94	(0.66, 1.31)	23	(8.1)	0.91	(0.55, 1.43)	16	(10.4)	1.62	(0.89, 2.78)
20–29 years	5615	(58.0)	482	(52.6)	1	(reference)	311	(49.9)	1	(reference)	171	(60.4)	1	(reference)	72	(46.8)	1	(reference)
30–34 years	2306	(23.8)	268	(29.3)	1.21	(1.03, 1.42)	199	(31.9)	1.34	(1.11, 1.62)	69	(24.4)	0.96	(0.72, 1.27)	41	(26.6)	1.16	(0.77, 1.73)
35–39 years	763	(7.9)	90	(9.8)	1.23	(0.96, 1.56)	70	(11.2)	1.41	(1.06, 1.86)	20	(7.1)	0.87	(0.52, 1.36)	18	(11.7)	1.45	(0.83, 2.42)
40+ years	115	(1.2)	10	(1.1)	0.86	(0.40, 1.63)	7	(1.1)	0.99	(0.41, 2.00)	3	(1.1)	0.62	(0.10, 2.00)	7	(4.5)	3.68	(1.48, 7.85)

a Relative risk and 95% confidence interval adjusted for birth year, gender, race and ethnicity, maternal age, gestational age and birth weight.

**Table 2:** Adjusted odd ratios for high birth weight and heavier maternal weight, acute lymphocytic leukaemia children born in New York State excluding New York City, 1988–2000

		**Controls**		**Cases (all ages)**				**Cases (ages 0–4 years)**				**Cases (ages 5–9 years)**			
**Birth weight (g)**	**Maternal weight (kg)**	***N***	**(%)**	***N***	**(%)**	**RR^a^**	**(95% CI)**	***N***	**(%)**	**RR^a^**	**(95% CI)**	***N***	**(%)**	**RR^a^**	**(95% CI)**
<3500	<80	2012	(51.1)	209	(44.3)	1.00	(reference)	157	(44.7)	1.00	(reference)	52	(43.0)	1.00	(reference)
<3500	80+	228	(5.8)	40	(8.5)	1.72	(1.16, 2.48)	32	(9.1)	1.72	(1.11, 2.60)	8	(6.6)	1.67	(0.72, 3.40)
3500+	<80	1404	(35.7)	192	(40.7)	1.26	(1.01, 1.57)	133	(37.9)	1.14	(0.88, 1.46)	59	(48.8)	1.65	(1.11, 2.46)
3501+	80+	294	(7.5)	31	(6.6)	0.99	(0.64, 1.48)	29	(8.3)	1.21	(0.77, 1.83)	2	(1.7)	0.16	(0.01, 0.75)

a Relative risk and 95% confidence interval adjusted for birth year, gender, race and ethnicity, maternal age and gestational age.
